# Epigenetic landscape in lysosomal storage disorders: mechanisms and modulation

**DOI:** 10.3389/fgene.2025.1679497

**Published:** 2025-11-11

**Authors:** Andrés Felipe Leal, Harry Pachajoa, Shunji Tomatsu

**Affiliations:** 1 Centro de Investigaciones en Anomalías Congénitas y Enfermedades Raras, Universidad Icesi, Cali, Colombia; 2 Centro de Investigaciones Clínicas, Fundación Valle de Lili, Cali, Colombia; 3 Nemours Children’s Health, Wilmington, DE, United States; 4 Institute for the Study of Inborn Errors of Metabolism, Faculty of Science, Pontificia Universidad Javeriana, Bogotá, Colombia; 5 Departamento de Genética Clínica, Fundación Valle de Lili, Cali, Colombia; 6 Faculty of Arts and Sciences, University of Delaware, Newark, DE, United States; 7 Department of Pediatrics, Thomas Jefferson University, Philadelphia, PA, United States

**Keywords:** epigenetics, histones, lysosomal storage disorders, methylation, miRNA

## Abstract

Lysosomal storage disorders (LSDs) are rare substrate-accumulating diseases primarily characterized by mutations in genes encoding proteins involved in lysosomal function, most of which have enzymatic activity. Resulting lysosomal dysfunction leads to the overaccumulation of non- or partially degraded substrates. While it is true that enzyme deficiency is the primary cause of LSDs, the epigenetic alterations in DNA methylation, miRNA expression, and histone modifications appear to be critical mechanisms involved in the pathogenesis of LSDs. As epigenetic marks are, in most cases, reversible, their study becomes vital to developing strategies aimed at reversing epigenome alterations. Although classical therapeutic alternatives aim to recover the lysosomal function by restoring the protein expression lost, the use of modifiers able to repair the epigenetic modifications in LSDs may become a promising strategy. This manuscript explores the most recent evidence on the epigenetic alterations in LSDs. It also discusses their modulation through epigenetic modulators, a novel and intriguing approach to treat LSDs, as well as the potential of the CRISPR/Cas9 system.

## 1 Introduction

Belonging to the inborn errors of metabolism (IEM), the lysosomal storage disorders (LSDs) encompass over 70 genetically inherited metabolic diseases characterized by the accumulation of undegraded substrates due to lysosomal dysfunction (Leal et al., 2020a; Ballabio, 2016; Parenti et al., 2021). LSDs are multisystemic pathologies, often presenting with neurological decline, skeletal abnormalities, hepatosplenomegaly, along with pulmonary and cardiac involvement (Parenti et al., 2021).

Resulting substrate accumulation leads to disturbances in cell homeostasis, which ultimately promote pro-oxidant, pro-inflammatory, and pro-apoptotic profiles (Leal et al., 2022). Beyond lysosomal substrate accumulation, as the primary cause of the LSDs, some studies suggest that epigenetic dysregulation may contribute to LSD pathogenesis (Hassan et al., 2017). For instance, aberrant DNA methylation, histone modification patterns, and disrupted non-coding RNA expression have been documented in LSDs (Fu et al., 2022; Shen et al., 2022; Morena et al., 2019; Vargas-López and Alméciga-Díaz, 2022; Xiao et al., 2019; Kunkel et al., 2023; Üzen et al., 2025; Tarallo et al., 2019; Tomatsu et al., 2005; Dasgu et al., 2015), further supporting their role in the pathomechanisms of these disorders.

Given that epigenetic modifications can be reversible, they emerge as attractive targets for therapeutic modulation (Lossi et al., 2024). Some molecules, such as GSK-J4, a histone demethylase inhibitor (KDMi) (Kunkel et al., 2023), RVX-208, which targets epigenetic readers (Fu et al., 2022), and miRNA overexpression (Inamura et al., 2021), have been evaluated in LSD models with encouraging results. Similarly, the use of histone deacetylase (HDAC) and DNA methyltransferase (DNMT) inhibitors already approved for use in oncology and neurodegeneration may also open a new avenue for rapidly moving into their repurposing for LSD treatment. The potential of these molecules is exciting, but equally thrilling is the use of advanced CRISPR/Cas9-based tools. This innovative strategy may open a promising opportunity for modifying epigenetic marks in LSDs.

In this manuscript, we review recent evidence on the role of epigenetic dysregulation in LSDs and the potential of treatments emerging from classical- and CRISPR/Cas9-based epigenetic mark-modifier approaches. The paper search was conducted using PubMed, Web of Science, and Google Scholar databases, and the following Boolean terms were applied: “lysosomal storage disorder” OR “LSD” AND “epigenetics” OR “dCas9” OR “epigenome” OR “DNA methylation” OR “histones” OR “chromatin”. Peer-reviewed papers from 2015 to 2025 were included, with a focus on those published between 2018 and 2025. Studies up to 2015 were systematically reviewed by Hassan et al. (2017), and we encourage interested readers to consult their work.

## 2 Molecular mechanisms of epigenetic signatures

### 2.1 DNA methylation

**FIGURE 1:**
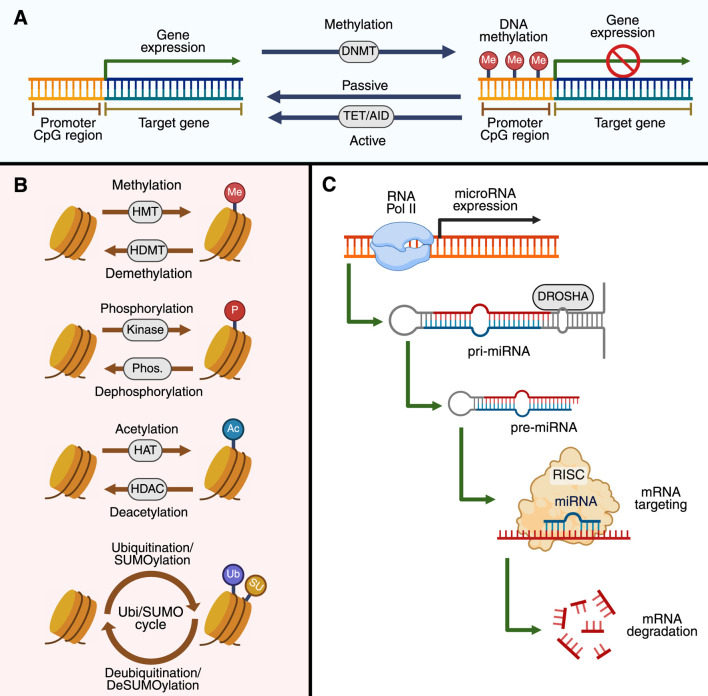
Molecular mechanism in epigenetics. In **(A)**, the processes of DNA methylation and demethylation control gene expression at CpG-rich promoter regions are shown. DNA methyltransferases (DNMTs) mediate the addition of methyl groups (Me) to cytosines, leading to gene silencing. Passive demethylation occurs during DNA replication, whereas active demethylation is catalyzed by the ten-eleven translocation (TET) enzymes and activation-induced cytidine deaminase (AID). In **(B)**, the post-translational modifications of histones are shown. Histone methyltransferases (HMTs) and histone demethylases (HDMTs) add and remove methyl groups (Me), respectively. Likewise, histones can also be phosphorylated (P) by kinases, while phosphorylation is removed by phosphatases (Phos.). Similarly, acetylation (Ac) of histones occurs through histone acetyltransferases (HATs) and is deacetylated by histone deacetylases (HDACs). Finally, histones can also undergo ubiquitination and SUMOylation (Ub/SUMO). In **(C)**, the mechanism of non-coding RNA expression is illustrated. DROSHA first processes primary microRNAs (pri-miRNAs) into precursor microRNAs (pre-miRNAs), which are then loaded onto RISC. In the RISC, mature miRNAs direct the complex to specific mRNAs, resulting in the degradation or translational inhibition of these mRNAs. The image was created by BioRender.com.

DNA methylation is mediated by the covalent addition of a methyl group to the 5′position of cytosine residues, typically within CpG-rich regions (Figure 1) (Mattei et al., 2022; Li and Tollefsbol, 2021). DNA methylation is regulated by cellular metabolism since it requires the methionine cycle to maintain the levels of S-adenosylmethionine (SAM), the universal source of the methyl group for DNA and histone methyltransferases (HMTs), and to control S-adenosylhomocysteine (SAH), a competitive inhibitor of HMTs (Bernasocchi and Mostoslavsky, 2024). Hypermethylation silences the transcription of genes by blocking the transcriptional machinery from binding to the genome; conversely, hypomethylation may result in the overexpression of genes (Mattei et al., 2022; Angeloni and Bogdanovic, 2019). DNA methylation is an essential physiological mechanism and a key epigenetic modification that underpins X-chromosome inactivation, genomic imprinting, and tissue-specific gene expression (Li and Tollefsbol, 2021). Aberrant DNA methylation is observed in pathological scenarios such as cancer (Li and Tollefsbol, 2021; Nishiyama and Nakanishi, 2021), autoimmune disorders (Ali et al., 2022; Danieli et al., 2024), neurodegenerative conditions (Ghosh and Saadat, 2023; De Plano et al., 2024), as well as some LSDs (Shen et al., 2022; Vargas-López and Alméciga-Díaz, 2022; Tomatsu et al., 2005). Importantly, oxidative stress has been documented in multiple LSDs (Ago et al., 2024; Lee and Hong, 2023; Leal and Alméciga-Díaz, 2022; Simoncini et al., 2020; Stepien et al., 2020) and may influence the activity of methyltransferases, thereby disrupting the SAM/SAH balance. Indeed, an increased accumulation of SAH can reduce the adequate availability of methyl donors, ultimately contributing to both global and gene-specific DNA hypomethylation. These findings support the idea that metabolic stress and impaired lysosomal function converge on the epigenome, linking classical metabolic dysfunction with transcriptional dysregulation in LSDs.

### 2.2 Histone modifications

Histones are basic proteins that package DNA, forming a structure known as nucleosomes (Knapp et al., 2023; Zhang et al., 2021). Their significance lies in their ability to undergo a variety of post-translational modifications (PTMs) at their N-terminal tails, such as acetylation, methylation, phosphorylation, ubiquitination, and sumoylation. These modifications play a pivotal role in shaping the chromatin structure and influencing gene expression (Figure 1) (Zhang et al., 2021; Millán-Zambrano et al., 2022). For instance, histone acetylation of lysine residues (e.g., H3K9ac) typically induces transcription by opening the chromatin. Conversely, certain methylation marks (H3K27me3, H3K9me3) are associated with transcriptional repression (Zhang et al., 2021; Jain and Epstein, 2024). The activity of histone acetyltransferases (HATs), histone deacetylases (HDACs), HMTs, and histone demethylases (KDMs), which contribute to chromatin dynamics, is tightly regulated at the transcriptional, post-translational, and cellular levels (Jain and Epstein, 2024; Ramaiah et al., 2021). The urgency of understanding histone modifications is further highlighted by their role in disease, with dysregulation of these processes being linked to several human diseases.

### 2.3 Non-coding RNAs

Non-coding RNAs (ncRNAs) form a vast and functionally diverse group of RNAs that are not translated into proteins but rather are key regulators of gene expression (Figure 1) (Nemeth et al., 2024; Chen and Kim, 2024). This diversity is evident in the fact that microRNAs (miRNAs), approximately 22 nucleotides in length, typically bind to complementary sequences in the 3′untranslated regions (3′UTRs) of target mRNAs, promoting mRNA degradation or translation repression. Similarly, long non-coding RNAs (lncRNAs), which are greater than 200 nt in length, are frequently implicated in chromatin modification, transcriptional interference, and post-transcriptional control via RNA stabilization or degradation (Chen and Kim, 2024; Virciglio et al., 2021). The complexity and diversity of ncRNA underscore their importance in molecular biology, and dysregulation of these molecules has been associated with several human diseases, including LSDs (Morena et al., 2019; Xiao et al., 2019; Üzen et al., 2025; Tarallo et al., 2019; Dasgu et al., 2015; Virciglio et al., 2021).

## 3 Epigenetics and LSDs

Recent evidence strongly supports that epigenetic dysregulation in LSDs may offer additional pathogenic mechanisms beyond the lysosomal accumulation. This underscores the promising potential of certain epigenetic signatures in the treatment, diagnosis, and prognosis of LSD patients. In this section, we delve into the latest developments in the field of epigenome alterations in LSDs, which have been succinctly summarized in Table 1.

**TABLE 1:** Main epigenetic findings in LSDs.

LSD	Epigenetic alteration	Physiological outcome	Ref.
Krabbe	↓miR-219	Oligodendrocyte dysfunction, impaired differentiation, increased apoptosis	Inamura et al. (2021)
GM2	↑/↓miRNAs (miR-9, -19a, −29a, −33, −34a, −124, −126a, −128, −137)	Brain region-specific miRNA profiles, neuroinflammation, disrupted lipid metabolism and synaptic function	Morena et al. (2019)
NPC	↓DNMT3a, ↓5mC, LINE-1 demethylation, ↓H3K27me3, ↓H3K9me3	Defective oligodendrocyte maturation, myelin gene dysregulation, increased apoptosis	Kunkel et al. (2023), Kennedy et al. (2016)
Gaucher	↓miR-15a, ↓miR-125b, ↑miR-21	Neurodegeneration, oncogenic risk, inflammation, subclinical shifts in carriers	Üzen et al. (2025)
Fabry	↓Methylation (AR promoter, COL4A1/2); sex-specific miRNA signatures (miR-19a-3p, miR-486-5p, let-7a, let-7d)	Proinflammatory signaling, therapeutic responsiveness in males	Shen et al. (2022), Xiao et al. (2019)
Pompe	↑miR-133a	Biomarker of severity and treatment response; elevated in infantile onset, reduced with ERT	Tarallo et al. (2019)
MPS IIIB	↓5mC, ↓H3K9me3, ↑H3K14ac (mutation-dependent)	Mutation-specific histone modification profiles, global hypomethylation	Vargas-López and Alméciga-Díaz (2022)
MPS IVA	Global DNA hypomethylation, H3K14ac increase in A393S (not in R94C/A393S)	Mutation-dependent chromatin states, preserved H3K9me3	Vargas-López and Alméciga-Díaz (2022), Tomatsu et al. (2005)

5hmC, 5-Hydroxymethylcytosine; 5mC, 5-Methylcytosine; AR, Androgen receptor; ERT, Enzyme replacement therapy; GM2, GM2 gangliosidoses; MPS, Mucopolysaccharidosis; NPC, Niemann-Pick disease, type C. ↓Downregulation. ↑Upregulation.

### 3.1 Krabbe

Krabbe disease (KD, OMIM # 245200) is an LSD caused by mutations in the *GALC* gene, leading to a deficiency of galactocerebrosidase (GALC) and accumulation of the glycolipids galactosylceramide (GalCer) and sulfatide (Maghazachi, 2023). GALC deficiency results in severe demyelination in the central nervous system (CNS), along with early-onset forms causing rapid neurodegeneration and early death (Maghazachi, 2023). Regarding epigenetics, Inamura et al. (2021) identified an epigenetic mechanism involving miR-219 in the oligodendrocyte pathology of KD using the twitcher mouse model (Inamura et al., 2021). The authors observed a significant decrease in the miR-219 expression in developing oligodendrocytes *in vivo* and *in vitro*. Interestingly, oligodendrocyte precursor cells (OPCs) isolated from twitcher mice failed to differentiate and exhibited increased caspase-3-mediated apoptotic cell death when cultured (Inamura et al., 2021). Likewise, functional assays using luciferase reporters confirmed that miR-219-mediated mRNA repression was diminished in KD oligodendrocytes (Inamura et al., 2021), suggesting that miR-219 downregulation contributes to oligodendrocyte dysfunction and demyelination in KD.

### 3.2 GM2 gangliosidoses

GM2 gangliosidoses are a group of three related LSDs, as follows: Tay-Sach (OMIM # 272800), Sandhoff (OMIM # 268800), and AB variant (OMIM # 272750), characterized by impaired degradation of the GM2 ganglioside (Leal et al., 2020b). The lysosomal accumulation of GM2 in neurons leads to progressive neurodegeneration, with early-onset forms typically causing motor decline, seizures, and early death in infancy or childhood (Leal et al., 2020b; Toro et al., 2021). A study reported by Morena et al. (2019) provided, for the first time, an in-depth analysis of miRNA dysregulation in mouse models of Tay-Sachs and Sandhoff diseases (Morena et al., 2019). This study conducted miRNA analysis of 12 candidates involved in lipid metabolism, neural development, and neuroinflammatory processes, specifically in the subventricular zone (SVZ) and the striatum region (STR) of the brain (Morena et al., 2019). Interestingly, the comparative analysis revealed distinct brain region- and disease-specific patterns of miRNA expression. Nine miRNAs (including miR-9, −19a, −29a, −33, −34a, −124, −126a, −128, −137) showed altered levels in SVZ and STR across models, with some miRNAs uniquely dysregulated in SVZ or STR (Morena et al., 2019). The computational miRNA-mRNA network analysis further predicted downstream effects on pathways central to lysosomal dysfunction, lipid trafficking, axon guidance, synaptic signaling, inflammation, and cell survival (Morena et al., 2019), suggesting that miRNA-mediated post-transcriptional regulation may play a critical, region-specific role in the epigenetic mechanisms of GM2 gangliosidosis.

### 3.3 Niemann-pick type C

Niemann–Pick type C (NPC) is an LSD caused by mutations in the NPC1 (NPC type C1, OMIM # 257220) or NPC2 (NPC type C2, OMIM # 607625) genes, leading to defective cholesterol and lipid transport out of lysosomes (Lee and Hong, 2023). Early studies conducted by Kennedy et al. (2016) in a murine model of NPC revealed DNA methylation disruption, accompanied by a significant decrease in DNMT3a and methyl-CpG binding protein expression, decreased 5-methylcytosine staining, global LINE-1 demethylation, and promoter-specific hypermethylation of single-copy genes, supporting the alteration of epigenetic markers (Kennedy et al., 2016). Most recently, Kunkel et al. (2023) have brought to light that NPC1 is essential for maintaining repressive histone methylation during oligodendrocyte maturation (Kunkel et al., 2023). In *Npc1* ^*−/−*^ mice, RNA sequencing revealed disrupted myelin gene expression and aberrant activation of neuronal programs in oligodendrocyte lineage cells, along with reduced numbers of OLIG2^+^ and MBP^+^ cells, increased apoptosis, and a loss of H3K27me3 and H3K9me3 marks (Kunkel et al., 2023), further uncovering pathogenic mechanisms in NPC.

### 3.4 Gaucher

Gaucher disease (GD) is an LSD characterized by a deficiency of the glucocerebrosidase (GCase), leading to the lysosomal accumulation of glucocerebroside. Clinically, three types of GD are distinguished as follows: type 1 (non-neuronopathic, OMIM # 230800), type 2 (acute neuronopathic, OMIM # 230900), and type 3 (chronic neuronopathic, OMIM # 231000) (Minervini et al., 2023). Epigenetic alterations, such as the dysregulation of miR-181c-5p, miR-34b-5p, and miR-10a-5p, have been identified in early studies using the neuronopathic GD mouse model and are attributed to the pathogenesis of GD by impairing axonal guidance, synaptic plasticity, and mitochondrial function (Dasgu et al., 2015). Altered miRNA profiles have been widely identified in cancer (Wilson and Scaffidi, 2025). Interestingly, a study conducted by Üzen et al. (2025) assessed the oncogenic miRNA expression profile in type I GD patients (Üzen et al., 2025). The blood-based profiling revealed significant downregulation of miR-15a and miR-125b, both known tumor suppressors (Üzen et al., 2025), and upregulation of the oncogenic miR-21 in patients undergoing enzyme replacement therapy (ERT) (Üzen et al., 2025), suggesting a sustained pro-oncogenic molecular state that could predispose GD patients to increased risks for multiple myeloma, hepatocellular carcinoma, and hematologic malignancies. Importantly, heterozygous carriers also exhibited reduced levels of miR-15a, miR-150, and miR-181b, indicating subclinical epigenetic shifts (Üzen et al., 2025). These findings significantly contribute to our understanding of GD, suggesting that epigenetic dysregulation may play a crucial role in neurodegeneration in GD and an increased oncogenic risk.

### 3.5 Fabry

Fabry disease (FD, OMIM # 301500) is an X-linked LSD caused by mutations in the *GLA* gene, leading to a deficiency of the α-galactosidase A (α-Gal A), resulting in pathological accumulation of globotriaosylceramide (Gb3) (Lenders and Brand, 2021). Lysosomal accumulation of Gb3 in FD patients particularly affects endothelial cells, the kidneys, the heart, and the CNS (Lenders and Brand, 2021). Epigenetic alterations in FD have been described in several studies. For instance, Shen et al. (2022) demonstrated that the α-Gal A defect leads to DNA hypomethylation, thereby modifying the methylation pattern of genes, including the androgen receptor promoter, and hypomethylation of the *COL4A1* and *COL4A2* genes, resulting in their overexpression (Shen et al., 2022). These findings were accompanied by elevated methionine levels in both patient-derived endothelial cells and FD mouse models (Shen et al., 2022), linking lysosomal glycosphingolipid accumulation to epigenetically driven transcriptional remodeling.

At the post-transcriptional level, several studies have revealed miRNA-based dysregulation in FD. For instance, Xiao et al. (2019) identified distinct serum miRNA signatures that correlate with treatment response (Xiao et al., 2019). Most importantly, as FD primarily affects males, the authors also included analysis based on sex differences. In this context, the levels of miR-19a-3p and miR-486-5p expression were significantly reduced in ERT-treated males compared to untreated males (Xiao et al., 2019), suggesting that an epigenetic response to therapy is quantifiable and measurable. Conversely, female patients showed greater diversity in the expression of the miRNAs (Xiao et al., 2019), possibly due to the larger clinical and molecular heterogeneity associated with X-linked transmission. These results suggest that individual miRNAs may serve as biomarkers to monitor treatment responsiveness in male Fabry patients, highlighting the importance of considering gender in epigenetic marker investigations. Other miRNAs predicting the occurrence of a proinflammatory process, such as let-7a and let-7d, were identified in FD (Maier et al., 2021), which may represent potential biomarkers. This discovery offers optimism for the future of disease monitoring in FD.

### 3.6 Pompe

Pompe disease (PD, OMIM # 232300) is an LSD caused by mutations in the *GAA* gene. *GAA* encodes acid α-glucosidase (GAA). GAA deficiency results in the progressive accumulation of glycogen (George et al., 2024). In PD, an analysis of miRNA conducted by Tarallo et al. (2019) in mouse models (at 3 and 9 months) and plasma from Pompe patients identified a specific miRNA profile associated with disease severity and treatment response (Tarallo et al., 2019). In mice, 211 miRNAs were dysregulated in skeletal muscle and 66 in the heart, with distinct patterns at different ages. Relevantly, in plasma from six patients, 55 miRNAs were differentially expressed, with 16 overlapping those altered in mouse tissues (Tarallo et al., 2019). The miR-133a was proposed as a key biomarker, with levels significantly elevated in Pompe patients, especially in infantile-onset cases, compared to late-onset cases, and correlating with clinical severity (Tarallo et al., 2019). Interestingly, in three infantile patients, miR-133a levels decreased upon ERT, coinciding with clinical improvement (Tarallo et al., 2019). These findings suggest that circulating miRNAs, particularly miR-133a, may serve as non-invasive biomarkers for monitoring disease progression and therapeutic efficacy in PD, offering hope for improved disease management.

### 3.7 Mucopolysaccharidosis IIIB

MPS IIIB (OMIM # 252920) is an LSD characterized by dysfunction of the lysosomal enzyme α-N-acetylglucosaminidase (NAGLU) (Rouse et al., 2024). Impaired NAGLU enzyme activity leads to the accumulation of heparan sulfate, primarily in the CNS (Rouse et al., 2024). Recently, Vargas-López et al. (2024) identified pronounced epigenetic alterations in two MPS IIIB patient-derived fibroblasts carrying different mutations in the *NAGLU* gene (Vargas-López et al., 2024). Although a global reduction in DNA methylation levels (5-methylcytosine) and the heterochromatin marker H3K9me3 was reported in both cell lines, the authors found that histone H3K14 acetylation was differentially altered in both MPS IIIB fibroblasts (Vargas-López et al., 2024). In fibroblasts carrying the P358L mutation, H3K14 acetylation was significantly increased. At the same time, it was found to be unaffected in E153K-containing MPS IIIB fibroblast models, supporting the notion that specific *NAGLU* mutations may differentially impact the epigenetic landscape in MPS IIIB.

### 3.8 Mucopolysaccharidosis IVA

MPS IVA (OMIM # 253000) is an LSD caused by mutations in the *GALNS* gene, which encodes for the lysosomal enzyme N-acetylgalactosamine-6-sulfatase (GALNS) (Leal et al., 2023). GALNS deficiency leads to the accumulation of keratan sulfate and chondroitin 6-sulfate, resulting in skeletal dysplasia and systemic manifestations in MPS IVA patients (Leal et al., 2023). Early studies conducted by Tomatsu et al. (2005) revealed methylation epigenetic alteration patterns at CpG dinucleotides in the *GALNS* gene (Tomatsu et al., 2005). Most recently, *in vitro* analyses of patient-derived fibroblasts have demonstrated a pattern of global DNA hypomethylation (Vargas-López et al., 2024). While the heterochromatin marker H3K9me3 remained preserved in MPS IVA, the H3K14 acetylation pattern in MPS IVA fibroblasts was found to be mutation-dependent, similar to that observed in MPS IIIB fibroblasts (Vargas-López et al., 2024). Remarkably, patients with MPS IVA harboring the A393S mutation had enhanced H3K14 acetylation. In contrast, the double mutation R94C/A393S did not affect the H3K14 acetylation profile (Vargas-López and Alméciga-Díaz, 2022), indicating that mutations can differentially influence epigenetic marks. These novel findings open new avenues for understanding and potentially treating MPS IVA.

## 4 Targeting epigenetic alterations in LSDs

Current evidence strongly supports the epigenetic dysregulation in several LSD. In consequence, seeking novel alternatives to restore the epigenetic alterations may offer a promising alternative for treating LSDs along with classical interventions, including ERT and GT. This last section explores classical and advanced approaches aimed at rescuing the altered epigenetic signature in LSDs.

### 4.1 Histone-modifying alternatives

The histone modification-based strategies rely on histone-modifying enzymes, including HDACs, HMTs, and KDMs (Yang et al., 2022). In NPC disease, loss of transcriptional silencing is observed, which impairs the proper function of oligodendrocytes (Kunkel et al., 2023). Notably, GSK-J4, a KDMi, has been reported to restore the maturation of NPC oligodendrocyte progenitor cells (Kunkel et al., 2023), supporting the concept that correcting the epigenetic signature could represent a valid therapeutic approach. Interestingly, HATi, such as garcinol, may be more suitable in conditions where histone hyperacetylation has been observed, for example, in MPS IIIB and MPS IVA, where specific mutations are associated with increased H3K14 acetylation (Vargas-López and Alméciga-Díaz, 2022). Taken together, these considerations highlight that the use of epigenetic modulators in LSDs should be individualized, guided by mutation-specific epigenetic signatures and the underlying chromatin contexts.

### 4.2 Epigenetic readers

Epigenetic readers are a class of proteins that bind to domains selectively recognizing covalent modifications on DNA, histones, and non-histone proteins deposited by epigenetic writers (Damiani et al., 2020). Upon binding, epigenetic readers recruit effector complexes that modify chromatin architecture and influence gene expression (Damiani et al., 2020). Consequently, targeting epigenetic reader proteins could be a promising alternative in epigenome modulation of LSDs. For instance, Apabetalone (RVX-208), which binds to the BD2 domain of the bromodomain and extraterminal-containing protein family (BET), has been shown to suppress proinflammatory transcriptional programs in innate immune cells from patients with FD undergoing ERT (Fu et al., 2022). Interestingly, RVX-208 led to a decrease in proinflammatory markers, including TNF-α, IL-12, MCP-1, and IL-6, as well as reduced oxidative stress (Fu et al., 2022), further supporting the notion that epigenetic modulators can positively impact the pathogenic features in LSD, such as FD.

### 4.3 microRNA-targeted approaches

miRNAs are the longest ncRNAs studied in LSD and are widely recognized to be altered (Morena et al., 2019; Xiao et al., 2019; Üzen et al., 2025; Tarallo et al., 2019; Dasgu et al., 2015). Since miRNA tightly regulates post-transcriptional gene expression of several factors involved in the pathogenesis of LSDs, such as lysosomal dysfunction, autophagy, immune activation, and oxidative stress, among others, miRNA are potential targets for treating LSDs. For instance, in KD, the downregulation of miR-219 has been identified as a key trigger of OPC dysfunction, which limits their maturation into oligodendrocytes (Inamura et al., 2021). Interestingly, treatment with exogenous miR-219 has shown potential in restoring maturation markers (MBP and PLP), while reducing caspase-3 activation and psychosine levels (Inamura et al., 2021), highlighting that targeting dysregulated miRNA may be an alternative for treating KD. Further research and clinical trials are necessary to fully explore the potential of miRNA-targeted approaches in LSDs; however, the initial results are promising.

In GD, miRNA profiling has consistently detected dysregulated miRNAs, which likely play a role in inflammation, synaptic dysfunction, and mitochondrial stress, ultimately contributing to the development of certain cancers (Üzen et al., 2025; Dasgu et al., 2015). Although there are no studies directly targeting miRNA in GD, the use of isofagomine, a pharmacological chaperone for GCase, normalizes approximately 40%–60% of altered miRNA in the brains of GD mouse models, suggesting that classical approaches may influence the epigenome in some LSDs (Dasgu et al., 2015). This finding underscores the potential of miRNA as a therapeutic target in LSDs, including GD, and warrants further investigation into the role of miRNA dysregulation in the pathogenesis of these diseases.

### 4.4 CRISPR/Cas9-based epigenome editing

**FIGURE 2:**
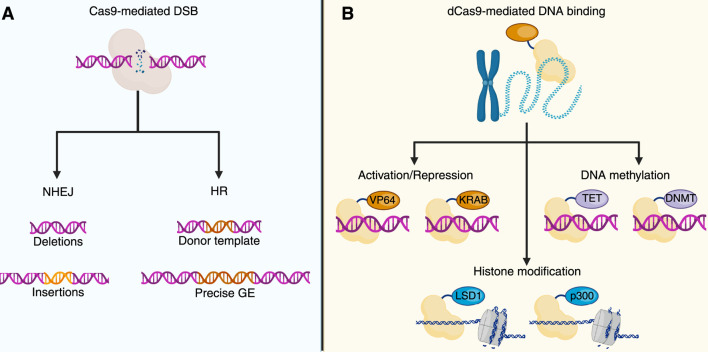
CRISPR/Cas and genome and epigenome editing. **(A)** CRISPR/Cas9 genome editing uses a catalytically active Cas9 that induces double-strand breaks (DSBs) at target loci. DSB repair by NHEJ may introduce small indels, while, in the presence of a donor DNA template, HR facilitates targeted genome modification. **(B)** CRISPR/Cas9-based epigenome editing (GE) utilizes a catalytically dead Cas9 (dCas9), which allows for the targeted recruitment of effector domains without inducing DNA cleavage. dCas9 fused to transcriptional activators (e.g., VP64) or repressors (e.g., KRAB) modulates gene expression. Joining with ten-eleven translocation enzymes (TET) or DNA methyltransferases (DNMT) allows locus-specific demethylation or methylation of the DNA. Histone modifications can also be mediated by dCas9-fused effectors, such as lysine-specific demethylase 1 (LSD1) or histone acetyltransferase p300. The image was created by BioRender.com.

Early reports demonstrated that the Clustered Regularly Interspaced Short Palindromic Repeats and CRISPR-associated protein 9 (CRISPR/Cas9) system is an immune system that protects prokaryotes from re-infection by phages (Barrangou et al., 2007; Jiang and Doudna, 2017). Later, studies conducted by Doudna and Charpentier demonstrated the suitability of CRISPR/Cas9 in recognizing, binding to, and cutting genomic DNA with high precision (Jiang and Doudna, 2017; Leal et al., 2024; Zhang, 2021), leading to development of a promising genome editing approach, which is currently used to induce knockouts and knock-ins (Figure 2), and resulting in its application for innovative gene therapies for treating human diseases (Sharma et al., 2021; Wan et al., 2023).

The CRISPR/Cas9 system includes a single guide RNA (sgRNA) that binds to a desired DNA sequence and recruits the Cas9 protein. Interaction of Cas9 with a protospacer adjacent motif (PAM) in DNA precedes sgRNA-mediated DNA binding (Jiang and Doudna, 2017; Jinek et al., 2012). Moreover, Cas9 introduces a double-strand break (DSB) in the DNA, ultimately promoting the activation of the DSB repair. Cas9 has two nuclease domains (HNH and RuvC) that generate the DSBs (Jiang and Doudna, 2017). If a donor template is co-delivered into the nucleus, homologous recombination takes place, and exogenous DNA information is inserted (knock-in). Instead, when no donor is available, the non-homologous end-joining (NHEJ) pathway is activated, introducing random insertions and deletions (indels), which can lead to knockouts (Leal et al., 2024; Chen et al., 2024). Interestingly, novel approaches have combined the extraordinary ability of CRISPR/Cas9 with the use of enzymes capable of modifying epigenetic signatures, resulting in a novel approach termed CRISPR/Cas9-based epigenome editing (EE) (Villiger et al., 2024; Fadul et al., 2023). This innovative approach utilizes the recognizing and binding properties of the CRISPR/Cas9 system while avoiding Cas9-mediated DSBs by employing a catalytically inactive Cas9 enzyme, also known as dead Cas9 (dCas9) (Cai et al., 2023). dCas9 carries two mutations in the HNH (H840A) and RuvC (D10A) domains that prevent DSBs within the DNA (Liu et al., 2020; Whinn et al., 2019). Along with dCas9, the CRISPR/Cas9-based EE leverages epigenetic effectors that can inhibit or activate gene transcription (Figure 2) (Koonin et al., 2023; Sar and Dalai, 2021). Table 2 summarizes common effectors used in CRISPR/Cas-based EE.

**TABLE 2:** Common epigenetic effectors evaluated in CRISPR/Cas9-based EE approaches.

Effector domain	Function	Target epigenetic mark	Mechanism	Ref.
DNMT3A	DNA meth	5 mC	Catalytic addition of methyl groups at CpGs	Park et al. (2022)
TET1	DNA demeth	5 mC → 5hmC → unmodified C	Oxidative removal of DNA methylation	Qian and Liu (2024)
KRAB	TR	Indirect (via H3K9me3)	Recruitment of SETDB1, HP1 to deposit repressive histone marks	Alerasool et al. (2020)
VP64	TA	Indirect (mediator recruitment)	Recruitment of transcription machinery	Omachi and Miner (2022)
p300 (HAT domain)	Hist. Ace	H3K27ac, H3K18ac	Acetyl transfer on histones at regulatory sites	Huang et al. (2023b); Wu et al. (2023)
LSD1 (KDM1A)	His. Demeth	H3K4me1/2, H3K9me1/2	Removes activating/repressive lysine methylation	Xie et al. (2018)
EZH2 (SET)	His. Meth	H3K27me3	Catalyzes trimethylation of H3K27	O'Geen et al. (2017)
G9a (EHMT2)	His. Meth	H3K9me1/2	Promotes heterochromatin formation	O'Geen et al. (2017)
SunTag	ESA	Configurable	Recruits multiple effector copies via repeating GCN4 epitopes	Albrecht et al. (2024)
SID4X	CR	Indirect (via HDACs)	Recruits mSin3/HDAC complexes	Carleton et al. (2017)
SAM (VP64 + p65+HSF1)	TA	Indirect (via histone modification)	Synergistic activator complex	Petazzi et al. (2024)

5mC, 5-methylcytosine; 5hmC, 5-Hydroxymethylcytosine; CR, Corepressor recruitment; DNA demeth, DNA demethylation; DNA meth, DNA methylation; DNMT3A, DNA methyltransferase 3 alpha; EHMT2, Euchromatic histone-lysine N-methyltransferase 2; ESA, Effector signal amplification; EZH2, Enhancer of zeste homolog 2; G9a, Euchromatic histone-lysine N-methyltransferase 2; H3K18ac, Histone H3 lysine 18 acetylation; H3K27ac, Histone H3 lysine 27 acetylation; H3K27me3, Histone H3 lysine 27 trimethylation; H3K4me1/2, Histone H3 lysine 4 mono-demethylation; H3K9me1/2, Histone H3 lysine 9 mono-/demethylation; His. Demeth, Histone demethylation; His. Meth, Histone methylation; Hist. Ace, Histone acetylation; KDM1A, Lysine demethylase 1A; KRAB, Krüppel-associated box; LSD1, Lysine-specific demethylase 1; p300, Histone acetyltransferase p300; SAM, Synergistic Activation Mediator; SID4X, Four tandem repeats of the Sin3 interaction domain; SunTag, Supernova tag system; TA, Transcriptional activation; TET1, Ten-eleven translocation methylcytosine dioxygenase 1; TR, Transcriptional repression; VP64, Tetrameric.

CRISPR/Cas9-based EE has been evaluated in several rare diseases, including Prader–Willi Syndrome (Rohm et al., 2025), Facioscapulohumeral Muscular Dystrophy (Mariot and Dumonceaux, 2022), and Fragile X Syndrome (Liu et al., 2018), among others. However, its true potential lies in its application to LSDs, where it has not yet been tested. Indeed, the epigenetic landscape observed in multiple LSD models strongly suggests that epigenome profile restoration may be within reach through CRISPR/Cas9-based EE, thereby offering a new avenue for treating LSDs (Figure 2).

## 5 Future perspectives

**FIGURE 3:**
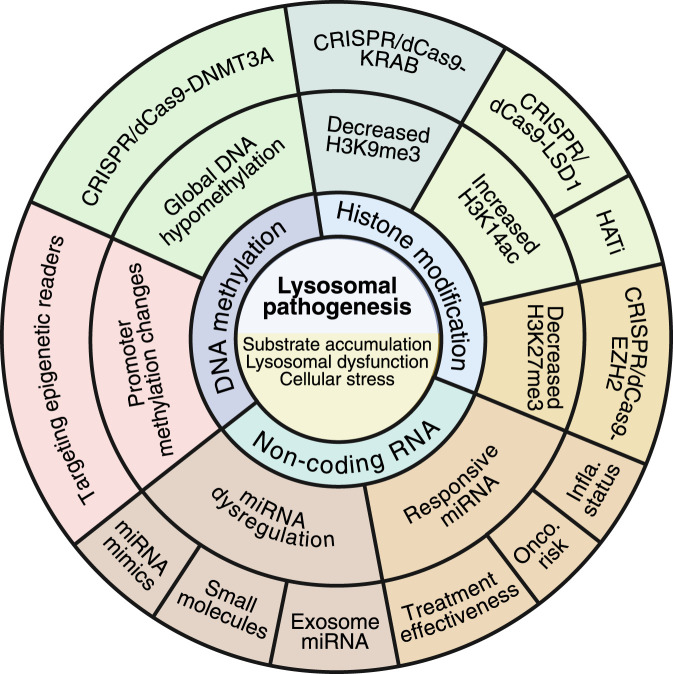
Kaleidoscope view of the epigenetic landscape in LSDs. The epigenetic landscape in LSDs has switched the classical view of these diseases from substrate-accumulating diseases to complex inherited metabolic disorders in which several epigenetic actors are actively contributing to the global cell homeostasis impairment. While epigenome alterations are gaining importance in the pathogenesis of LSDs, novel therapeutic alternatives are emerging to rescue these alterations. Likewise, some epigenetic marks have been recognized as playing a role in monitoring disease progression, prognosis, and oncogenic risk. The image was created by BioRender.com.

The understanding of the epigenetic landscape in LSDs provides a new paradigm in the LSD pathogenesis, from monogenic, substrate-accumulating diseases to complex disorders involving transcriptional and chromatin-level dysregulation, which contribute to the disturbance of cell homeostasis. Importantly, as epigenetic modifications can be reversed, several therapeutic strategies hold enormous potential to ameliorate the epigenetic alterations in LSDs (Figure 3). In this regard, the use of HAT inhibitors, epigenetic readers modulators, miRNA mimics, or antagonists may provide a further direction in treating LSDs. Beyond pharmacological approaches, the interplay between metabolism and the epigenome opens new opportunities for nutritional and metabolic interventions in LSDs. Strategies aimed at supporting one-carbon metabolism, including supplementation with methyl donors such as folate, choline, or betaine, may help restore the SAM/SAH ratio and partially correct DNA hypomethylation (Bernasocchi and Mostoslavsky, 2024; Inoue-Choi et al., 2012). Evidence from metabolic and neurodegenerative disorders suggests that such interventions may improve epigenetic homeostasis (Bekdash, 2021; McKee et al., 2017; Araki et al., 2022). Although these approaches remain largely unexplored in LSDs, metabolic adjuvants could represent safe, accessible, and potentially synergistic options to complement ERT, GT, or emerging epigenetic treatments.

Novel studies have addressed the use of exosomes for transporting miRNA in non-LSD (Huang et al., 2023a), which may also constitute a promising alternative in the treatment of LSDs. Indeed, the large number of FDA-approved molecules may accelerate the implementation of epigenetic drugs into the classical approaches used in LSDs. Likewise, the latest advances in the CRISPR/Cas9 system, a revolutionary tool for editing the epigenome, may offer a novel and exciting strategy for restoring epigenetic disturbances in LSDs, thereby increasing the treatment options available.

Finally, the use of epigenomic signatures may benefit early diagnosis, monitor therapeutic response, and classify patients, especially in LSDs such as Gaucher and Fabry, where epigenetic perturbations may influence oncogenic risk or inflammatory status (Figure 3). However, it is crucial to emphasize the importance of systematically investigating sex-specific epigenetic variation and mutation-dependent chromatin states, recently highlighted in Fabry and MPS subtypes. These variations could be key factors in the progression and prognosis of LSDs, making this research an urgent and significant area of study.
